# Serum complement system activation in normal healing and atrophic non-union of human long bone fractures

**DOI:** 10.3389/fimmu.2026.1825939

**Published:** 2026-06-09

**Authors:** Yasser M. El-Sherbiny, Youssif M. Ali, Elena Jones, Peter V. Giannoudis, Jehan J. El-Jawhari

**Affiliations:** 1 Department of Biosciences, School of Science and Technology, Nottingham Trent University, Nottingham, United Kingdom; 2 Clinical Pathology Department, Faculty of Medicine, Mansoura University, Mansoura, Egypt; 3 Department of Veterinary Medicine, Cambridge Veterinary School, University of Cambridge, Cambridge, United Kingdom; 4 Leeds Institute of Rheumatic and Musculoskeletal Medicine, School of Medicine, University of Leeds, Leeds, United Kingdom

**Keywords:** classical pathway, complement system, fracture healing, atrophic non-union, lectin pathway (MBL)

## Abstract

**Introduction:**

The complement system has an important role in physiological bone healing, as studied mainly in animal models and local bone tissues. However, the role of the complement system in human fractures, particularly at the systemic level, remains insufficiently characterized. This study aimed to investigate the activation levels of the serum complement system during three healing phases (inflammation, repair and remodeling) of normal healing of long bone fractures and in patients diagnosed with fracture non-union.

**Methods:**

Blood samples were obtained from two groups of long bone fracture patients (normal healers and non-union) and healthy controls with no fractures. Blood samples from patients with normally healed fractures were collected at 1 week, 1 month, and 4–6 months post-fracture. Blood samples from patients diagnosed with non-union were collected at 1-year post-fracture. The serum samples were processed using mass spectrometry to quantify the complement protein expression. The ELISA was used for validation. Ingenuity Pathway Analysis (IPA) software was used for molecule-pathway interactions.

**Results:**

The classical complement pathway components, complement C1s and C1r, were significantly increased during the inflammation phase of normally healed fractures but reduced subsequently. The levels of serum C3, C3a, and C9 were significantly greater in the inflammatory phase than in the other phases. No significant differences were observed for other complement pathway components, complement factor B (CFB), complement factor H and I (CFH, CFI), ficolin 2 and 3 (FCN2, FCN3) and mannan-associated serine protease-1 (MASP1) when comparing the three phases. In fracture non-union, the serum MASP1 level was significantly higher than that of normal fracture healers and healthy controls. The IPA analysis showed a link between MASP1 as a part of the lectin pathway and damage in bone and cartilage.

**Discussion:**

Collectively, our data indicate temporal changes of the serum complement system with activation via the classical pathway, particularly during the inflammatory phase of normal healing of human bone fractures. Furthermore, systemic MASP1 levels were high in non-united fractures, indicating that the lectin pathway plays a unique role in abnormal fracture healing. This data will offer fresh avenues for utilizing complement system components, such as follow-up biomarkers and therapeutic targets, in bone injuries and diseases.

## 1 Introduction

Fracture healing is a physiological regenerative process that restores the damaged bone to its normal structure without scar formation in three phases (1). The early inflammatory phase occurs within a few hours of the fracture and lasts up to 1 week. A hematoma develops near the fracture site, leading to the recruitment of inflammatory cells, including lymphocytes, monocytes, macrophages, and polymorphonuclear cells, to the injury site. Consequently, granulation tissue will be formed, vascular tissue will grow, and mesenchymal stem cells (MSCs) will migrate to the fracture site (2). A fibrocartilaginous callus characterizes the repair phase, then the chondrocytes differentiate terminally, leading to hypertrophy and producing a mineralized cartilage matrix (3). The cartilaginous callus will be replaced by the bony callus, resulting in a more stable structure (3). The late remodeling phase involves restoration of the bone original structure and strength via balancing bone formation by osteoblasts and bone resorption by osteoclasts. While most fractures progress to normal healing, approximately 5-10% unfortunately fail to unite, resulting in fracture non-union. The development of fracture non-union is multifactorial, and although the lack of blood supply, severe injuries, bone instability and infections can delay healing, it is unknown why atrophic non-union occurs without these predisposing factors (4).

The complement system is part of the innate immune system, and it is activated via three complement activation pathways: the classical, the lectin, and the alternative pathways (5). The classical pathway is typically triggered by immune complexes and involves activation of the complement 1r (C1r) enzyme, which in turn activates C1s to cleave C4 and C2 to form the C3 convertase (6). The lectin pathway is typically initiated by the binding of the carbohydrate recognition molecules, mannose-binding lectin (MBL), collectin-10, collectin-11, ficolin-1, ficolin-2, and ficolin-3 to the surface of pathogen-associated molecular patterns (PAMPs) or damage-associated molecular patterns (DAMPs) (7). The binding of lectin recognition molecules to their target sites results in the activation of the MBL Associated Serine Proteases (MASP1 and 2) to cleave C4 and C2, leading to the formation of C3 convertase (8). Unlike the other two pathways, the alternative pathway is initiated by spontaneous hydrolysis of C3, resulting in the binding of Factor B (CFB), which is cleaved by Factor D (CFD) to form the C3 convertase (9–11). Activation of any of the three pathways of the complement system involves the C3 cleavage by C3 convertase, which produces C3a and C3b. Additionally, this activation shifts the activity of the convertases toward C5, forming C5 convertases, and the terminal pathway (lytic) of complement activation is initiated by the cleaving of C5 via C5 convertase to C5b and C5a. The C5b molecule will deposit on the cell membrane, followed by the deposition of C6, C7, and C8. The complex of C5b-8 molecules recruits multiple C9 molecules, forming Membrane Attack Complexes (MAC) (12).

Activation of the complement system has been reported to participate in the healing process of various damaged tissues, with the complement cascade activation causing neutrophil migration into the wound site to remove apoptotic cells and eliminate infection, helping to heal the wound subsequently (13). Furthermore, it has been shown that the complement system activation following anterior cruciate ligament injury and meniscal tear contributes to the development of post-traumatic osteoarthritis (14). Zhang et al. demonstrated that skeletal muscle regeneration after injury is associated with early activation of the complement system (15). The role of the complement system in bone fracture healing has been mainly studied at the bone injury site and in animal models of fracture. The expression levels of C3aR and C5aR1 were significantly increased throughout the healing process of fractures in bone cells (16). In addition, reduced callus volume and mechanical stability were observed in C5-deficient mice (17). In human studies, high complement activation was reported in elderly individuals, particularly those with osteoporosis, indicating a therapeutic potential (18), but this potential is unclear for traumatic long bone fractures. Additionally, no studies have tested which activation pathways in the complement system are triggered, particularly at the circulatory level and in human normal-healed fractures.

In the herein study, complement levels were quantitatively measured in serum samples of patients with long bone fractures, aiming to assess the systemic activation of the complement pathways during the three phases of physiological fracture healing. The serum expression of complement was also assessed in the diagnosed non-union fracture group, compared to the remodeling phase of normal fracture healers and healthy controls. Identifying such immune clues in circulation can provide new means to monitor healing and understand immune mechanisms in bone healing complications.

## 2 Methods

### 2.1 Participants

The study protocols were reviewed and ethically approved with NREC number 06/Q1206/127, Humber-Leeds East, and the National Research Ethics Committee Yorkshire. Written consent was obtained from all the participants (a total of 33: 21 fracture patients, and 12 healthy controls) involved in this study before sample collection.

All patients in this study had traumatic long bone fractures (tibia: 14, femur: 3, humerus:3, and ulna:1). The normally healed fractures group (n=11) was defined clinically (lack of pain and mobility) and radiologically (computed tomography scans or on-plane radiographs) as healed at 4–6 months. The fracture non-union (n=10) was defined by the lack of radiographic signs of healing after nine months of fracture and with continuing pain at the non-union site during activity. All non-united fractures were atrophic, i.e. no infection. Patients were excluded if they had a pathological (non-traumatic) fracture, cancer, an immune disorder, diabetes, or bone metabolic diseases. Healthy controls (n=12) were defined as having no fractures or other diseases. The age of fracture patients and healthy controls was not significantly different (mean of 56 (range 30-74) and 55 (range 25-75) years old for non-union and normally healed fractures, respectively, and 47 (range 27-60) years old for healthy controls, with a gender match (male: female ratio was 7:4 or 7:3 for fractures and 7:5 for healthy controls).

### 2.2 Blood samples

Peripheral venous blood samples (10 ml) were taken from normal fracture healers within 1 week after the bone fracture, corresponding to the inflammatory phase (U1). A second blood sample was taken after 1 month (U2) for the repair phase, and the third was after 4 to 6 months post-fracture, indicating the remodeling phase (U3). Blood samples were collected from non-union patients at the time of diagnosis (a mean of 22 ± 8 months post fracture). As a control, blood samples were collected from healthy controls. The blood tubes were centrifuged at 2000 g for 10 minutes to separate the serum. The serum samples were aliquoted and stored at -80 °C till used for analysis.

### 2.3 Mass spectrometry

Serum samples were subjected to overnight trypsinization in 0.1% formic acid with a filtration at 0.22 μM. The samples were then desalted and cleaned up using HyperSep™ C18 Cartridges, then dried using a speed vacuum. The samples were then resuspended in 0.1% formic acid and 5% acetonitrile. The samples were then analyzed using a TripleTOF^®^ 6600 Quadrupole Time-Of-Flight (QTOF) mass analyzer for ESI-LC-MS/MS, SCIEX. The solid phase, a 15cm YMC Triart-C18 column, was used. The mobile phases for RP-HPLC were solvents A and B, which included 0.1% (v/v) FA and 2% (v/v) LC/MS grade ACN in LC/MS grade water, respectively. Scitex SWATH^®^ was used to acquire each sample measurement, which was then isolated and run against a protein library created from a pool of all the samples in a Data Dependent Acquisition (DDA/IDA). With 100 variable SWATH acquisition windows, the LC gradient for the IDA acquisition was conducted over an 87-minute gradient in high sensitivity mode, and the DIA over a 57-minute gradient (25 ms accumulation). For the data processing and analysis, retention durations were synchronized in Peak View and exported to Sciex Oneomics. Protein Pilot version 5 was used to generate data. The number of peptides having a “Peptides (95%)” value was determined to be connected to that protein with a 95% confidence level using DIA-NN 1.9.2 software.

### 2.4 ELISA

Complement component C1s, C1r, MASP1, C3a and C5a ELISA kits were used per the manufacturer’s manual (ThermoFisher Scientific and R&D Systems). The standards and samples were diluted as per the optimized conditions and incubated for 2.5 hours, and the ELISA plate was washed. In the next step, the biotin conjugate solution was added to the wells and incubated for 1 hour. Then, Streptavidin-HRP solution was added to the wells and incubated for 45 minutes, and the plate was washed. Substrate solution was added to wells, and the plate was incubated for 30 minutes in the dark. The stop solution was added, and the color change of the plate was read at 450 nm.

### 2.5 IPA software analysis

Ingenuity Pathway Analysis (IPA) was used to identify molecular relationships, associated diseases, and biological functions. The findings were mapped onto the Ingenuity Knowledge Base, a curated database derived from published experimental literature and based on documented biochemical, regulatory, or physical associations (19). Direct interaction indicates binding, cleavage, or phosphorylation. Indirect interaction reflects an intermediate−mediated relationship, in which many interactions occur through multi−step signaling cascades. Disease and Function analysis identified biological processes and bone pathologies that were statistically associated with changes in specific MASP1 and complement gene/protein levels.

### 2.6 Statistical analysis

The statistical analysis and graphs were prepared using GraphPad Prism 7. The Shapiro-Wilk normality test was used to select the comparative tests between groups. The comparative tests were conducted based on group numbers, the normality test, and whether the groups were paired. When the p-value < 0.05, the results were estimated as significant.

## 3 Results

### 3.1 The serum proteins show distinctive changes during phases of normal fracture healing

**Figure 1:**
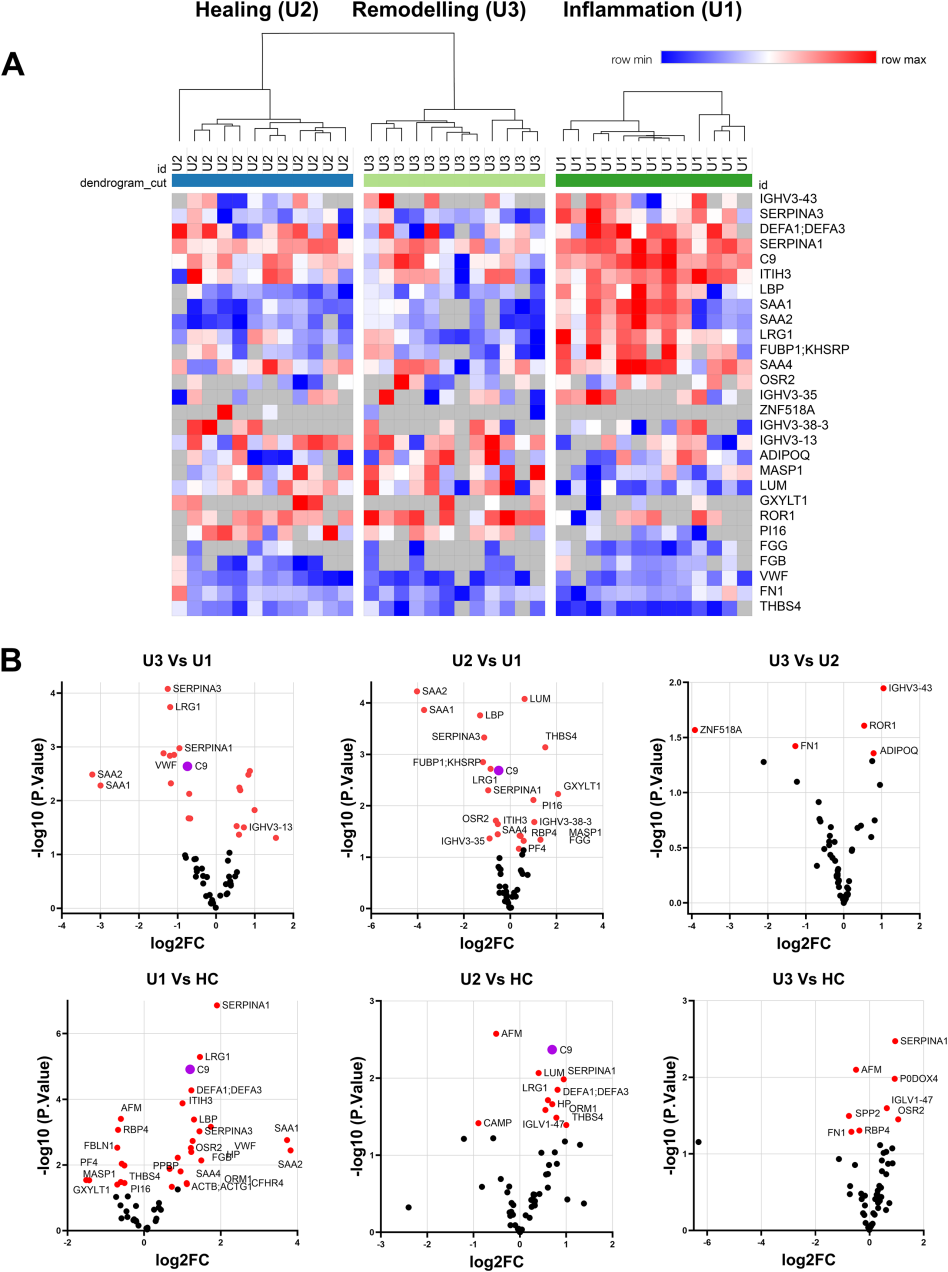
Mass spectrometry data for serum samples from healed long bone fractures and no-fracture controls. The heatmap shows the distinctive clustering of three phases of bone healing **(A)**. The volcano plots for two group comparisons. The red dots indicate significantly changed proteins, and C9 is shown in purple **(B)**. The inflammatory- 1 week post fracture (U1), the repair- 1 month post fracture (U2), the remodelling-4–6 months post fracture (U3) phases, and healthy no-fracture controls (HC).

Mass spectrometry data showed that serum proteins with statistically significant differences were clustered distinctly across three phases of bone healing: inflammatory (U1), repair (U2), and remodeling (U3), as shown in the heat map (**Figure 1A**).

The differential analysis was conducted using volcano plots to compare the phases of bone healing (**Figure 1B**), which showed that some serum proteins were higher or lower in the inflammatory phase than in the repair and remodeling phases. There were 30 proteins that differed between the inflammatory (U1) and repair (U2) phases. Altered expression of serum proteins was also noted between the inflammatory (U1) and remodeling (U3) phases (32 proteins). Only 11 serum proteins differed between the repair (U2) and remodeling (U3) phases. These results showed that the inflammatory phase had the most significant alteration in serum proteomics compared to the other phases of bone healing. The largest changes noted in serum proteins during the inflammatory phase appear to decrease as bone healing progresses.

The volcano plots (**Figure 1B**) also showed that the number of serum proteins differed between the inflammatory phase (U1) and no-fracture healthy controls (HC), which was the highest, followed by those showing differences between the repair phase (U2) and HC. The least number of altered serum proteins was noted when comparing the remodeling phase (U3) and HC. This data further indicated that the initial changes during the inflammatory phase of bone healing are gradually reduced through the phases until they become comparable to those of no-fracture control serum.

Among proteins that were found to be greatest in the inflammatory phase and gradually reduced in the repair phase, C9 was consistently detected in U1 vs. U3, U1 vs. HC, and U2 vs. HC (**Figure 1B**). This indicates that complement system activation occurs at a systemic level during the healing process of long bone fracture healing.

### 3.2 The classic complement pathway is activated systematically during the inflammatory phase of bone healing

**Figure 2:**
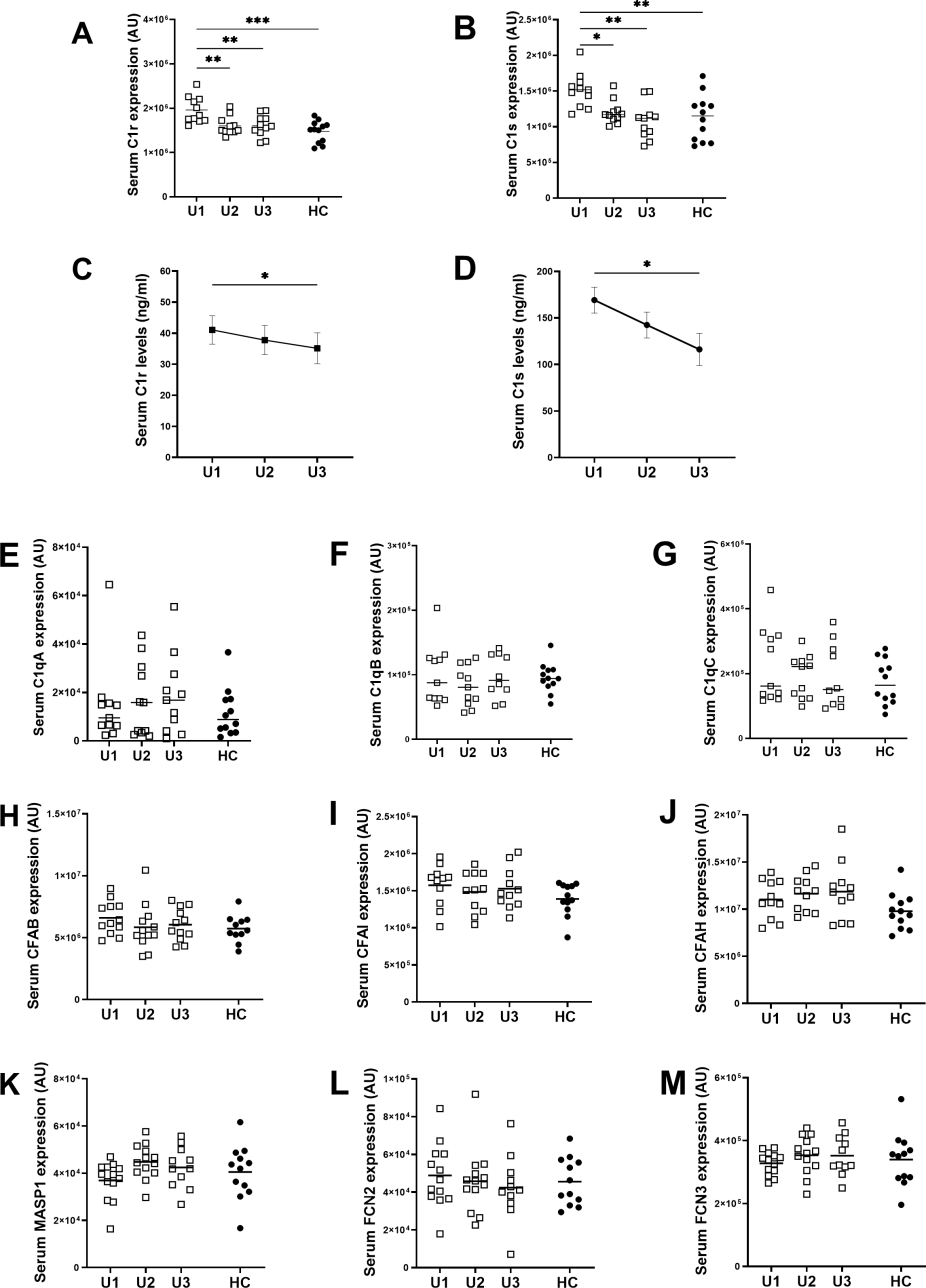
Systemic levels of three complement pathways in the serum of normal fracture healers. Serum expression levels of the classical pathway components, C1r and C1s, using mass spec **(A, B)**. Confirmed levels of C1r and C1s using ELISA **(C, D)**. Serum expression levels of C1qA, C1qB and C1qC **(E–G)**. Serum expression levels of the alternative pathway **(H–J)**. Serum expression levels of the lectin pathway **(K–M)**, n=11 for normal healed fractures (U1, inflammation/1 week post fracture; U2, repair/1 month post fracture; U3, remodeling/4-6-month post fracture), n=12 for healthy controls (HC). AU, arbitrary units for mass spectrometry. *p-value ≤0.05, **p-value <0.01, ***p-value <0.001.

As the differential proteomics analysis showed that serum C9 was physiologically induced in response to bone fractures, we examined the components of the classical, lectin, and alternative pathways that could activate the complement system. The mass spectrometry data showed that the serum levels of the classical pathway components, C1s and C1r, were significantly elevated during the inflammatory phase of bone healing compared to the levels measured during the repair, remodeling, and no-fracture healthy control groups (**Figures 2A, B**). To further substantiate the detected changes, the protein levels of C1r and C1s were measured using ELISA. The data confirmed that both C1r and C1s levels were significantly higher in early inflammatory healing than in the later phases of normal healed fractures and healthy controls (**Figures 2C, D**), confirming systemic induction of the classical pathway at the early phase of normal fracture healing. In contrast to C1s and C1r, the levels of C1q proteins (A, B and C) showed no significant differences (**Figures 2E–G****).**

Unlike the classical pathway, no changes were detected in the systemic levels of the alternative pathway components, Factors B (CFB) and I (CFI), throughout the phases of bone healing, as measured by mass spectrometry (**Figures 2H, I**). Interestingly, we observed that the regulatory factor complement factor H (CFH) exhibited a higher expression throughout the healing phases compared to controls. However, these differences were not statistically significant (**Figure 2J**). These data indicate no systemic signs of involvement in the alternative pathway during complement activation throughout fracture healing. Furthermore, we investigated the levels of the lectin pathway components in serum during the phases of normal fracture healing and healthy controls. The serum levels of mannan-associated serine protease 1 (MASP1) and Ficolin 2 and 3 (FCN2 & FCN3) remained unchanged in the three phases of fracture healing and were like those of healthy controls (**Figures 2K–M**). These data overall confirm that the complement classical pathway shows distinctive changes in the serum during the inflammatory phase.

### 3.3 High serum levels of complement anaphylatoxin C3a and activated lytic pathway were detected during the inflammatory phase of bone healing

**Figure 3:**
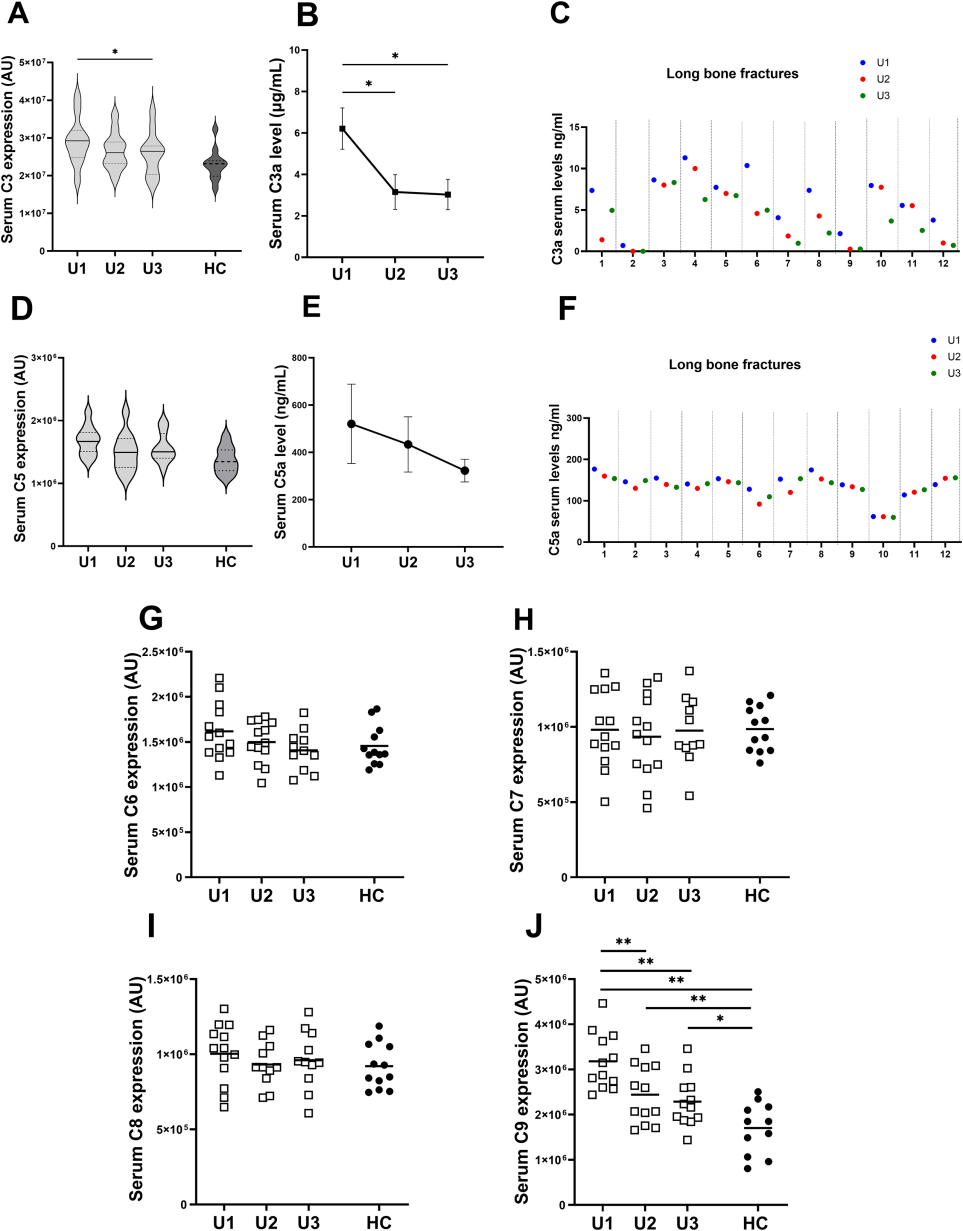
Systemic levels of C3, C5, anaphylatoxins and lytic pathway components during normal fracture healing. Serum expression levels of C3 and C3a, using mass spectrometry **(A)** and ELISA for collective and individual normal fracture patients **(B, C)**. Serum levels of C5 and C5a using mass spectrometry and ELISA **(D–F)**. Serum expression levels of the lytic pathway components, C6, C7, C8, and C9, as determined by mass spectrometry **(G–J)**, n=12 for normal healed fractures (U1, inflammation/1 week post fracture; U2, repair/1 month post fracture; U3, remodeling/4–6 month post fracture), n=12 for healthy controls (HC). AU, arbitrary units for mass spectrometry. *p-value ≤0.05, **p-value <0.01.

As the activation of the classical pathway in serum during the early inflammatory phase of bone fracture healing was activated, the upstream and downstream markers of the complement system activation were quantified in serum. Total serum C3 levels were significantly higher in the early inflammatory phase compared to those in the repair phase, and significantly higher than in the remodeling phase (**Figure 3A**). Additionally, the levels of the products of cleaved C3 and C3a were measured using ELISA. Interestingly, the serum levels of C3a were significantly higher during the inflammatory phase of the healing process compared to the repair and remodeling phases, and this was seen collectively and in individual patients (**Figures 3B, C**). In contrast, C5 levels have not shown differences between the three phases (**Figure 3D**). Similarly, no significant changes were detected in C5a levels for the collective or individual patients (**Figures 3E, F**).

Next, we tested whether activation of the terminal lytic pathway can be detected at the systemic level by measuring the components of the membrane attack complex. The levels of C6 and C7 in serum samples were similar during the three bone healing phases and comparable to those of healthy controls (**Figures 3G–I**). In contrast, the expression levels of serum C9 levels were highest during the inflammatory phase, followed by the repair phase, and during modelling, and all were significantly higher than healthy levels (**Figure 3J**). These data collectively indicate that, due to systemic classical complement pathway activation, serum C9 remains elevated until the remodeling phase.

### 3.4 Systemic complement activation is detected in the diagnosed atrophic non-union fractures with high MASP1 levels

**Figure 4:**
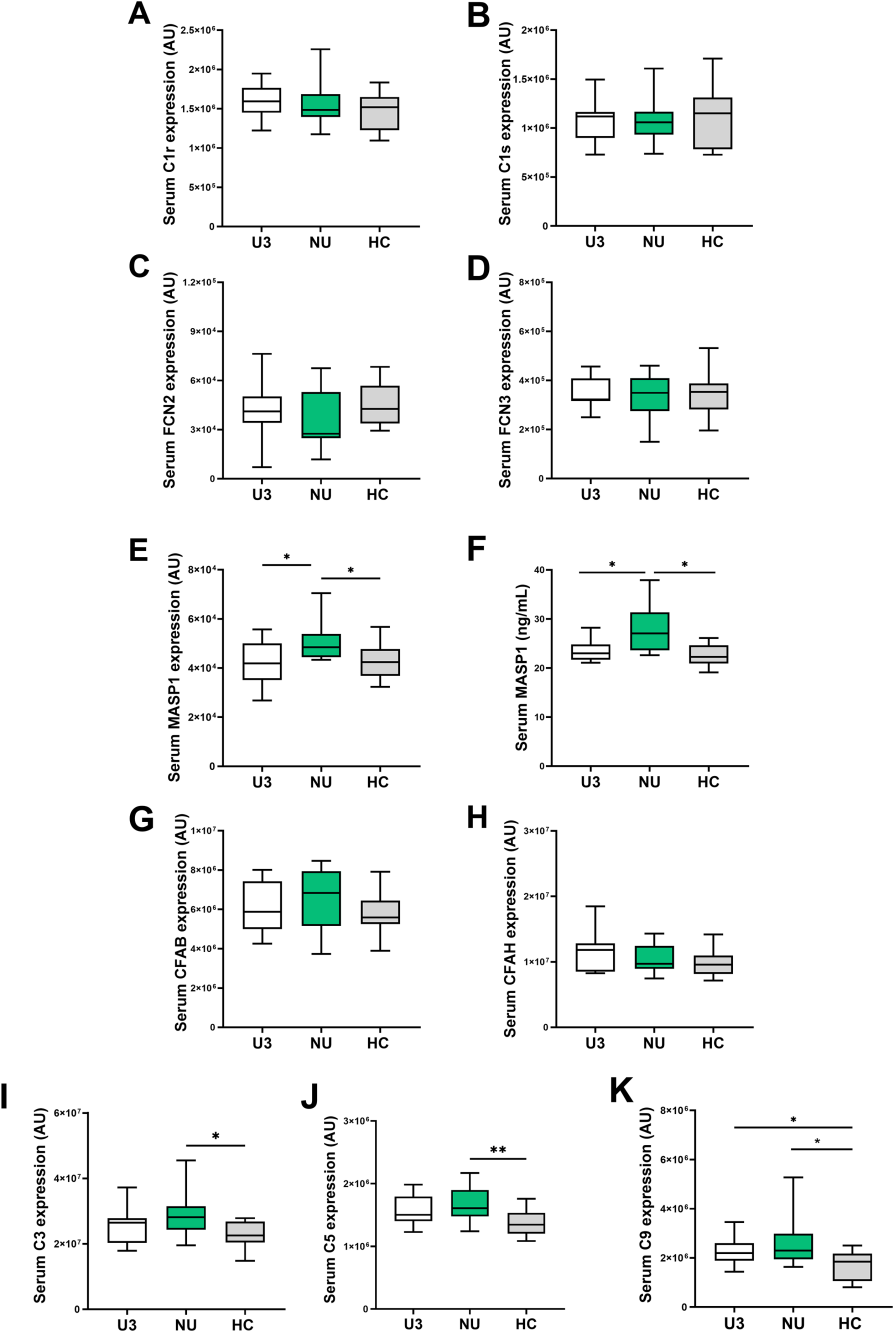
Systemic complement levels in atrophic non-union fractures. Serum levels of the classical pathway components C1r and C1s **(A, B)**. Serum expression levels of the lectin pathway components, FCN2 and FCN3 **(C, D)**, and MASP1 expression levels **(E)**, measured by mass spectrometry and confirmed by ELISA **(F)** and alternative pathway components, CFAB and CFAH **(G, H)**. Serum C3, C5 and C9 levels **(I–K)** measured by mass spectrometry, n=11 for normal healed fractures (U3: remodeling/4-6-month post fracture, white boxes), n=10 for non-union (NU: > 9 months post fracture, green boxes), and n=12 for healthy controls (HC, grey boxes). AU: arbitrary units for mass spectrometry. *p-value ≤0.05, ** p-value <0.01.

The activation of the classical complement pathway was systematically detected during the inflammatory phase of physiological bone healing, with C9 remaining persistently high until the remodeling phase. To assess whether any systemic signs of complement activation could be linked to complicated bone healing, a group of atrophic non-united fracture serum samples collected at diagnosis (a year or more after injury) was compared to the remodeling phase of normal fractures and healthy control groups. The mass spectrometry data showed that the serum levels of complement, C1s and C1r, the components of the classical pathway in the non-union group, were not different between those of healthy controls and the remodeling phase of normally healed fractures (**Figures 4A, B**), indicating a similar response for this pathway between non-union and normal healed fractures. Interestingly, while FCN2 and FCN3 levels have no differences between the groups (**Figures 4C, D**), the MASP1 serum levels were higher in the non-union fracture group than those of the normal healed fractures and healthy control groups, as measured by both mass spectrometry and ELISA (**Figures 4E, F**), demonstrating specific activation in non-union fractures. There was no association between MASP1 levels and sample collection times (**Supplementary Figure 1****).** Components of the alternative pathway, CFB and CFH, showed no differences between the groups (**Figure 4G, H**).

The serum levels of total C3 and C5 in fracture non-union were like those of normally healed fractures, but significantly higher than those of healthy control subjects (**Figures 4I, J**). Serum C9 levels in non-united fractures were like those of the remodeling phase of the normally healed fracture, but both groups had significantly higher C9 than those of normal controls (**Figure 4K**). These data collectively indicate an abnormal, persistent systemic activation of the complement system in the diagnosed fracture non-union, with higher MASP1 levels in these non-united fractures.

### 3.5 Complement cascade activation by MASP1 is linked to bone and cartilage damage

To understand why MASP1 is highly activated in non-union fractures, an analysis of the database of Ingenuity Pathway Analysis (IPA) software was conducted with a network centered on MASP1 and its molecular interactions within the complement cascade and the downstream bone-related biological functions and diseases, potentially influenced by these interactions.

**Figure 5:**
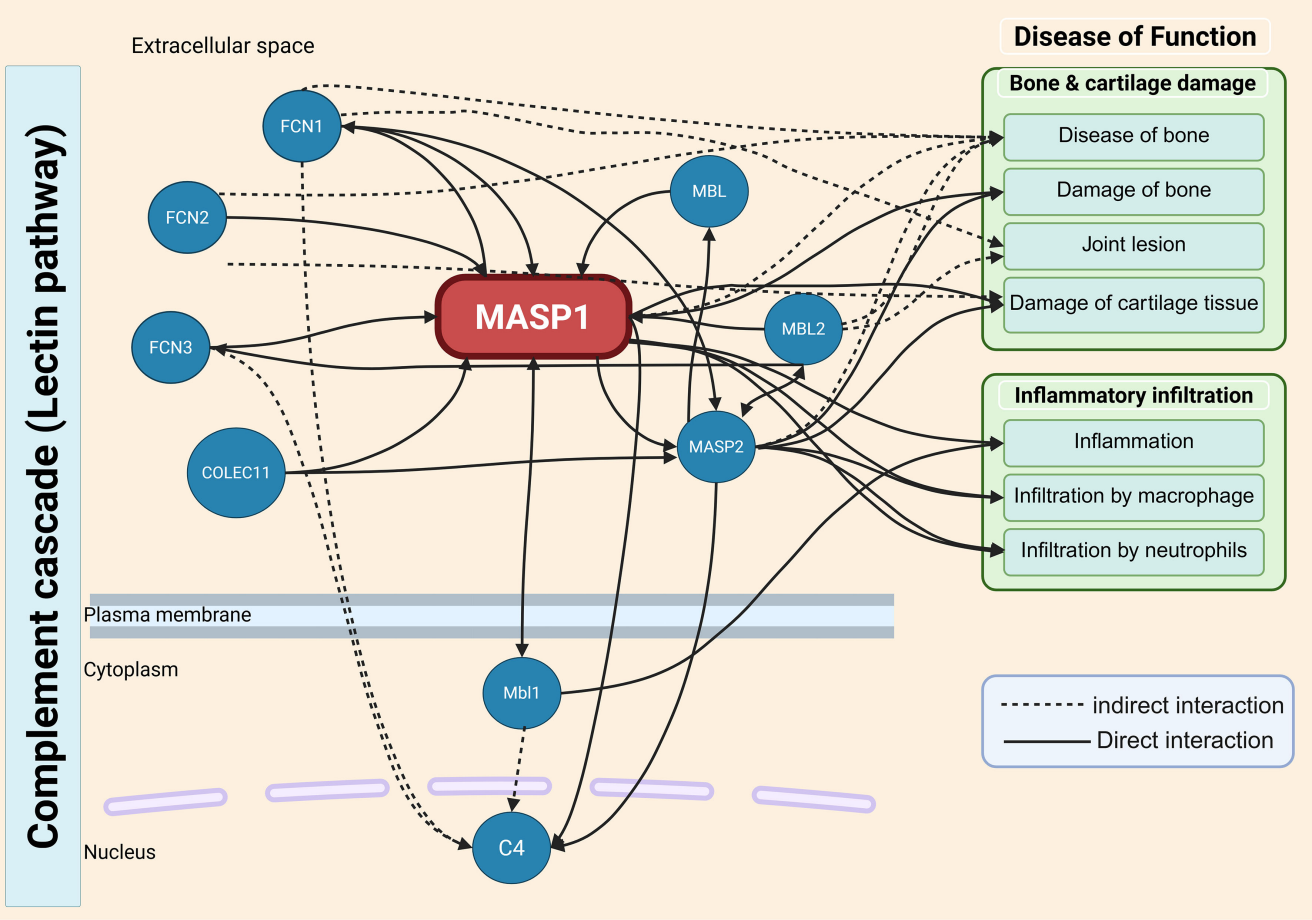
Interactions of MASP1 with lectin pathway components and its link with bone biology processes. Serum MASP1 interacts with other complement cascade components of lytic and alternate pathways and is known to be linked to bone and cartilage damage. Activation of MASP1 is predicted to increase damage to bone and cartilage via MASP2 and MBL proteins. Solid and dashed lines indicate direct and indirect interactions, respectively. Source: the Qiagen IPA software database, and the image was assembled in https://BioRender.com.

The data highlighted MASP1 as a key upstream regulator within the lectin pathway of complement activation. MASP1 interacts with FCN1, FCN2, FCN3, MASP2, COLEC11, and MBLs, which are all located predominantly in the extracellular space, except MBL1, which is expressed in the cell cytoplasm (**Figure 5**). This indicates how MASP1 interacts with these pattern−recognition molecules to initiate activation of the complement cascade.

The results also show MASP2, another lectin−pathway serine protease that functions in connection with MASP1. Both MASP1 and MASP2 connect downstream to complement component C4, located near the plasma membrane region (**Figure 5**), reflecting their known role in the lectin pathway, leading to complement activation.

Importantly, the data show links between MASP1 and related lectin-pathway molecules and bone-related disease and functions (**Figure 5**), suggesting that dysregulation of MASP1−associated pathways may influence bone healing in non-union fractures. Additionally, functional links were detected between MASP1 and other lectin pathway components on one side and neutrophil and macrophage recruitment and inflammatory activation (**Figure 5**), confirming the classic role in inflammation processes. Overall, MASP1 is a pivotal mediator bridging recognition molecules of the lectin pathway with complement activation and a range of downstream inflammatory and bone damage processes.

**Figure 6:**
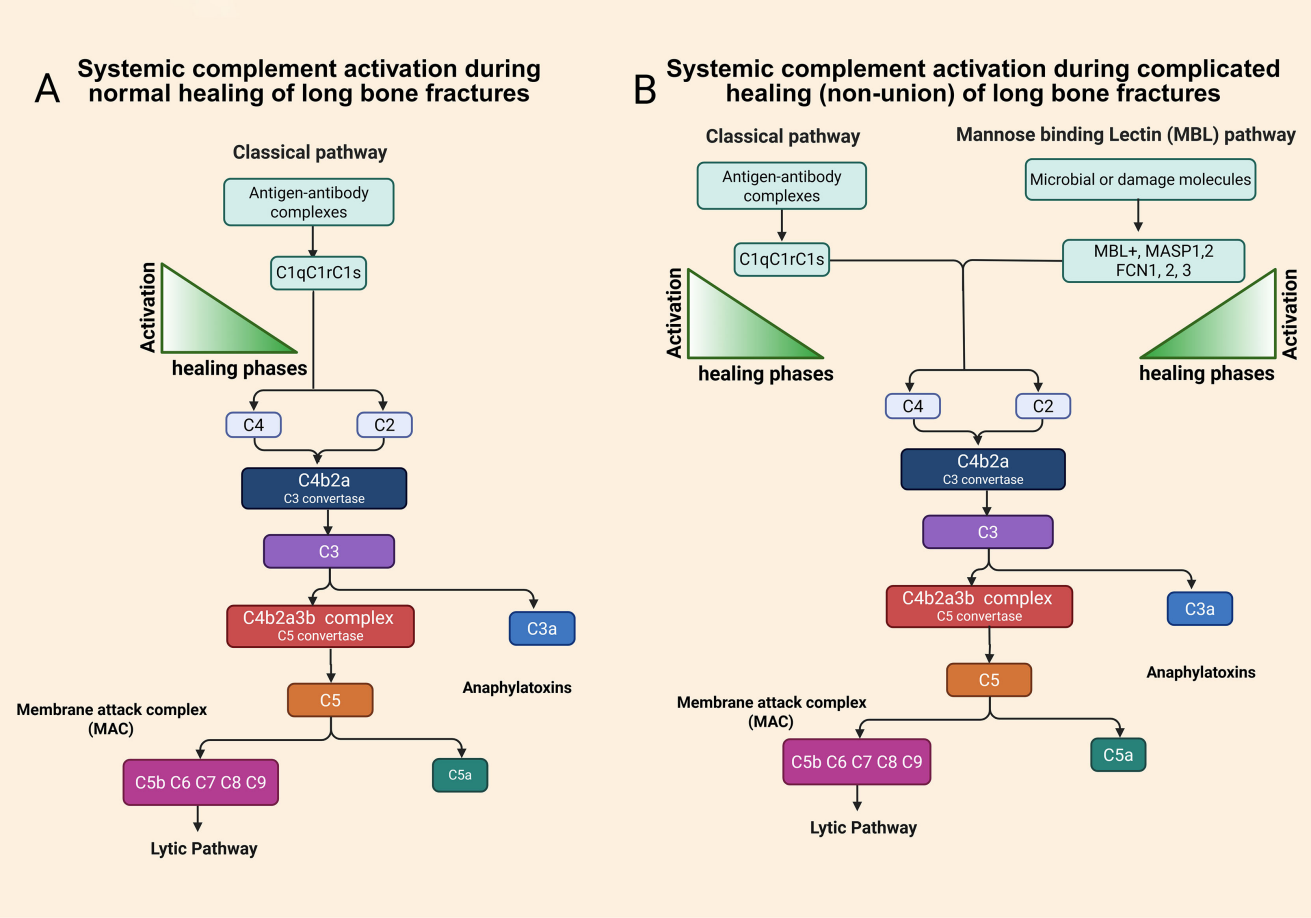
Graphical summary: Systemic complement activation in long bone fractures. In normally healed fractures, systemic activation of complement is mainly initiated early by the classical pathway, with progressive reduction till the remodeling phase **(A)**. In the atrophic non-union fractures, a similar early systemic activation of theclassical pathway is proposed, with late systemic complement activation due to activation of the lectin pathway **(B)**. Created in https://BioRender.com.

Collectively, the study findings indicate that in normal fracture healing, systemic activation of the complement system is mainly activated early by the classical pathway (**Figure 6A**). In the non-united fracture, no activation of the classical or alternative pathway was observed at the diagnosis (late remodeling). Only late systemic complement activation was noted to be associated with high MASP1 levels (**Figure 6B**), indicating a late activation of the lectin pathway in the non-union long bone fractures.

## 4 Discussion

This study aimed to unravel the expression levels of the complement system in the blood during normal healing of human long bone fractures. Additionally, we tested whether there were systemic alterations of complement activation in non-united fractures with no infection (atrophic non-union). Bone healing is a complex biological process involving immune, stromal and regenerative players during three phases, i.e., the inflammatory phase, the repair phase, and the remodeling phase (2). The complement system is an important initiator and amplifier of inflammatory responses and is involved in the process of bone turnover in some diseases. Complement protein abnormalities, including C1r, C2, C4, C3 and mannose-binding lectin (MBL), have been reported in systemic lupus erythematosus (SLE), who frequently display bone-density loss (20). Furthermore, C3 has proven to have a role in regulating bone turnover, particularly associated with osteoporosis (18, 21). While the complement system effect on the healing of fractures was shown in animal studies (16, 17, 22), its role in human fractures is limitedly known. In the present study, we uniquely showed the systemic activation of the complement during the inflammation phase of physiological fracture healing associated with classical pathway involvement. Additionally, our data proved a systemic signature of lectin pathway activation in atrophic non-union fractures.

Herein, it was found that the classical complement pathway components, C1s and C1r, are induced at the blood level during the inflammatory phase of normal bone healing, followed by a reduction at the repair and remodeling phases. Unlike the induced serum levels of C1r and C1s during the inflammation phase, no changes were detected in C1q levels. Differences in cellular source, biological function, and post−translational regulation may explain these findings. C1q is primarily synthesized by myeloid cells, particularly macrophages, rather than hepatocytes, unlike C1r and C1s (23). Additionally, C1q acts as the recognition component of the C1 complex and has a phagocytosis role, whereas C1r and C1s are serine proteases responsible for initiating downstream complement activation. C1q binds mainly to apoptotic cells and damage−associated molecular patterns (DAMPs) molecules at the sites of injured tissues (24), consequently, it may be consumed locally during immune complex clearance, potentially masking systemic changes in its levels. It is also possible that abnormality in C1 inhibitor (C1-INH) can contribute to these variable expression levels in C1 proteins, as C1-INH can selectively regulate C1r and C1s without directly affecting C1q (25). Consistent with our findings, previous studies have shown that changes in C1r, C1s, and C1q expression are not necessarily coupled. For example, patients who are deficient in C1r or C1s can have normal C1q levels (6, 26). Moreover, tumor cells have been reported to activate the classical complement pathway through excess production of C1r and C1s, but not C1q (27).

To our knowledge, our study is the first to report the specificity of systemic classical pathway activation during the inflammation phase in long bone traumatic fractures. The classical pathway is typically initiated by antigen-antibody complexes, but in the context of trauma, it may be triggered by damage-associated molecular patterns (DAMPs) released from necrotic or stressed cells. These DAMPs can bind to C1q, the recognition molecule of the classical pathway, thereby initiating the complement cascade (6). The classical pathway is preferentially activated late in an immune response to allow inflammation to wind down in a regulated manner and promote clearance and repair, unlike lectin and alternative pathways that have a lesser role in regulating inflammation and a greater contribution to pathological healing (28). Although we did not capture the individual peak time of inflammation for each patient, which might be variable, we assessed systemic levels of complement proteins during the overall inflammatory period (within one week post−injury). Notably, apart from one patient, all our inflammatory samples were collected within ≤ 4 days. The complement system activation can persist for up to 6 days post-fracture, as demonstrated in the ankle bone fractures (29). Additionally, in polytrauma patients, serum levels of the complement activation products or regulator components have been reported to remain elevated for up to 10 days post−injury (30, 31). We also acknowledge that trauma severity may influence the extent of complement activation. However, as all fractures were isolated, injury severity data were not available, and our analyses focused on paired comparisons across time points within the same individuals rather than between different patient groups, it is unlikely that variability in trauma severity significantly affected our temporal findings.

No changes in the lectin or alternative pathway were detected during the three phases of normal bone fracture healing. The lectin pathway is activated when one of several recognition molecules, MBL, collectins, or ficolins, bind to carbohydrates, e.g., on a pathogen surface, and foreign body (32), leading to elimination of apoptotic cells by the innate immune system (22). The alternative pathway activation was found to be activated in major trauma with brain and soft tissue injuries (33). Both lectin and alternative pathways can be promoted through the binding to PAMPs or DAMPs. As the long bone fractures included in this study were isolated fractures and had no infection, this might explain no activation signs for lectin and alternative pathways, at least at systemic levels. Additionally, the absence of activation of alternative and lectin pathways during normal healing suggests a highly selective immune response and a regulated strategy to limit excessive inflammation and tissue damage.

We also detected higher C9 levels in the inflammatory phase that reduced over time, likely due to classical complement pathway activation. The lytic pathway consists of C5–9 proteins, forming a Membrane attack complex (MAC), which helps fight against infections. C9 was not studied before for bone injuries, but it was reported to be persistently high in serum samples after 6 months from traumatic brain injuries (34). Since our data shows a significant increase in serum C9 levels compared to healthy controls till remodeling phase, C9 can also be a potential biomarker indicating the progress for physiological fracture healing.

We detected that during the initial phases of fracture healing, C3a level increases in blood as compared to the repair and remodeling phases. As the most abundant component of the complement system, C3 serves as a convergent site for all three activation pathways and is degraded into at least two fragments, C3b and C3a (35). In comparison to systemic levels, C3a may be more active at the local bone site. The expression of the C3a receptor is induced at the femur fracture site of the C57BL/6 mouse model during the healing period (36). Additionally, C3a recruits circulating immune cells, further amplifying the inflammatory process. C3a, present in injured tissues, also contributes to the recruitment of mesenchymal stem cells, inducing both immune response and tissue regeneration (12, 37). These roles show the importance of the production of C3a during the inflammatory phase to facilitate the transition to the repair phase. A previous study showed that C3a action on osteoclasts mediates the bone loss and osteoporosis mouse model (18, 38). These studies indicate that C3a is needed for the early phase of healing, as C3a can mediate excess bone resorption via osteoclasts, causing pathological bone loss or abnormal remodeling. In agreement, we found a reduction of serum C3a during the third phase (remodeling), where osteoclasts are most active in adjusting the healed fractured bone, likely to keep the balanced bone resorption.

No changes were noted for systemic levels of C5a, which might indicate a limited role as a biomarker in the healing process. C3a and C5a are both anaphylatoxins, but the generation of C3a is approximately 300 times faster than the generation of C5a (36), and this might explain differences in systemic expression. Also, our serum findings do not conflict with the C5 role in local bone tissues, particularly for the remodeling phase. C5a mediates osteoclastogenic effects via several effects on macrophages, osteoclasts and immune cells (39–42). C5-deficient mice displayed a reduced volume and mechanical properties in the fracture callus, indicating impaired healing (16). C5aRs are strongly expressed in the fracture callus by immune cells, bone cells and chondroblasts (16). C5aR1 knockout mice showed a decrease in early inflammation in the fracture callus and disturbed healing (43).

Our data showed that serum MASP1 was increased in diagnosed fracture non-union compared to the remodeling phase of normally healed fractures or healthy controls. MASP1 is one of the lectin pathway components, and its high levels in non-union might indicate persistent tissue stress leading to sustained complement activation. This finding agrees with research showing that chronic inflammation and immune dysregulation are common features in bone damage. A link between the excess complement role, e.g. factor H-related protein-5 (CFHR5), and pathological bone formation in ankylosing spondylitis has been shown (44). At the fracture site and during the inflammatory phase, the lectin pathway activation helps clotting factors to hold immune and mesenchymal stem cells within the fracture hematoma (22, 45). Also, the lectin pathway was reported to have a role in enhancing the clearance of dead cells by macrophages, so it is likely to have a role in the bone remodeling phase (22, 46). However, for systemic expression, the only study by de Seny et al. demonstrated that serum levels of C3, C4 and C6 are upregulated compared to healthy individuals (47). While that study did not detect MASP1, it interestingly reported a significant serum reduction of ITIH4 (Inter-alpha-trypsin inhibitor heavy chain 4), which acts as a regulator of the lectin pathway by directly inhibiting MASP1 and MASP2 (48). It is possible that the differences in fracture characteristics (i.e., having long and flat bone injuries in de Seny et al. study compared to only long bones in our study), different timepoint measurements (at 19 months versus our study at 22 months), and the non-inclusion of any normal fracture healers could be reasons for not detecting MASP1 changes. Nevertheless, both our and de Seny studies indicate directly or indirectly the lectin pathway activation at a systemic level in facture non-union. Systematic MASP1 level serves as an immune-related biomarker in sepsis and positively correlates with the severity of the brain trauma (49). Although biomarkers are of great value in predicting healing, few markers have been introduced in human studies assessing the progress of traumatic fracture healing. Therefore, quantitative measurement of serum MASP1 could be considered as a prognostic tool for non-union of a fracture and may help plan treatment.

The IPA analysis importantly shows that MASP1 can be a link between innate immune recognition and inflammatory effector responses, leading to tissue damage. Two MASPs (MASP1, MASP2) are the key initiators of the lectin pathway of the complement cascade. After binding to MBL, they recognize, cleave, and activate complement proteins C2 and C4, generating the complement convertase C4b2a. Cleavage of the complement proteins C3 and C5 produces C3a and C5a, two pro-inflammatory anaphylatoxins that cause degranulation of inflammatory cells (including mast cells and phagocytes), and induce an inflammatory response at the site of complement activation. Previous studies have linked MASP/MBL proteins to bone damage. MASP1 and MASP3 serum levels were shown as potential biomarkers predicting new bone formation among axial spondylarthritis patients (50, 51). Another study showed that mutations in the MASP1 and MASP3 genes were associated with lumbosacral bone anomalies (52). Furthermore, serum MASP1 levels have been reported to be significantly reduced in osteoporosis and to correlate with low bone mineral density in postmenopausal women (53). Additional links to joint or damage diseases, such as rheumatoid arthritis (RA) or osteoarthritis, were also reported. Inhibition of MASP1 was associated with a reduction in C3 deposition and synovial macrophage and neutrophil infiltration in a mouse model of RA (54). Previous research has also shown that MASP1/3 KO mice were resistant to arthritis, highlighting the role of MASP1/3 in developing inflammatory tissue injury (55).

The serum levels of MASP2 or MASP3 were not measured due to the limited availability of serum samples. We focused on quantifying MASP1 level, given its important role as an inducer of the lectin pathway and being the primary activator of MASP2 (56). MASP1 and MASP3 are splice variants transcribed from the same gene, but they have different functions. MASP1 activates the lectin pathway, while MASP3 regulates the alternative pathway (57).

A *post hoc* power analysis was also conducted for the comparison of MASP1 between non-union and the controls, which showed a significant change (p-value ≤0.05). This analysis indicated adequate statistical power (≥ 0.80) with a sample size of 10–12 patients per group. Therefore, our observed findings are unlikely to be influenced by sample size limitations.

For the non-union samples, we also acknowledge the wide range in sample collection times, which partly reflects variability in clinical referral timelines. Therefore, we analyzed the MASP1 levels against the sample collection times. To address this, we examined the relationship between MASP1 levels and sample collection time. This analysis revealed no clear association between MASP−1 levels and the timing of sample collection.

Together, these data support the value of MASP1 as a novel systemic marker that could specifically predict abnormal remodeling in atrophic non-union of long bones. Future research is needed to confirm the prognostic value of this biomarker in a larger cohort of atrophic non-union fractures, also in bone remodeling diseases such as osteoporosis, particularly with recent research showing that reducing C3 protect from osteoporosis in a mouse model of ovariectomy (18). Additionally, it would be of interest to explore the potential role of MASP1 in other forms of complicated fracture healing, such as infection or hypertrophic complications. In case of bone infection, such as osteomyelitis, three complement activation pathways are known to be involved (17). MASP1 has also been proposed as a marker for monitoring trauma severity and sepsis, owing to its ability to activate the IL−6/JAK−STAT3 signaling pathway (49). Additionally, there is evidence linking complement activity to hypertrophic bone responses, as a polymorphism in complement factor H has been associated with a reduced risk of heterotopic ossification (58). However, whether the MASP1/MBL pathway plays a specific role in these types of complicated fracture healing remains to be investigated.

In summary, our study provides evidence that the classical complement pathway is selectively activated in patients with normal healed long bone fractures at a systemic level. These findings offer new insights into the immunological landscape following acute skeletal trauma, which may reflect regulatory mechanisms of immune interactions unique to bone tissue. Future studies should explore the temporal dynamics of complement activation across different types of traumas or in polytrauma patients. Additionally, mechanistic studies are needed to elucidate the precise triggers and regulatory checkpoints that govern this selective complement pathway activation. Our study also reveals that serum MASP1 levels are significantly elevated in patients with atrophic non-union fractures compared to those undergoing normal fracture healing. This finding adds to other immune disturbances that lead to abnormal bone healing. In the rodent fracture model, elevated circulating levels of immune inhibitory cells, MDSCs, and IL-10 can also predict poor functional healing outcomes (59). For human fractures, the induction of blood MASP1 levels may have significant implications for both monitoring and therapeutic interventions. Differential levels of complement proteins have been previously shown to be involved in the development of post-traumatic osteoarthritis (PTOA) after anterior cruciate ligament injury (ACL) and meniscus tear (MT) injuries (14). Serum complement levels as a non-invasive diagnostic tool during fracture healing phases could provide a forward-looking angle, relevant for clinicians and researchers alike.

## Data Availability

The original contributions presented in the study are publicly available. This data can be found here: https://doi.org/10.5281/zenodo.18945873.

## Supplementary material

Supplementary Figure 1 MASP1 levels against the collection times of non-union fracture samples.
