# Supplementary figures and images for: Serum complement system activation in normal healing and atrophic non-union of human long bone fractures
Source: Front Immunol. 2026 Jun 9;17:1825939. doi: 10.3389/fimmu.2026.1825939 (PMC13286776; doi:10.3389/fimmu.2026.1825939)

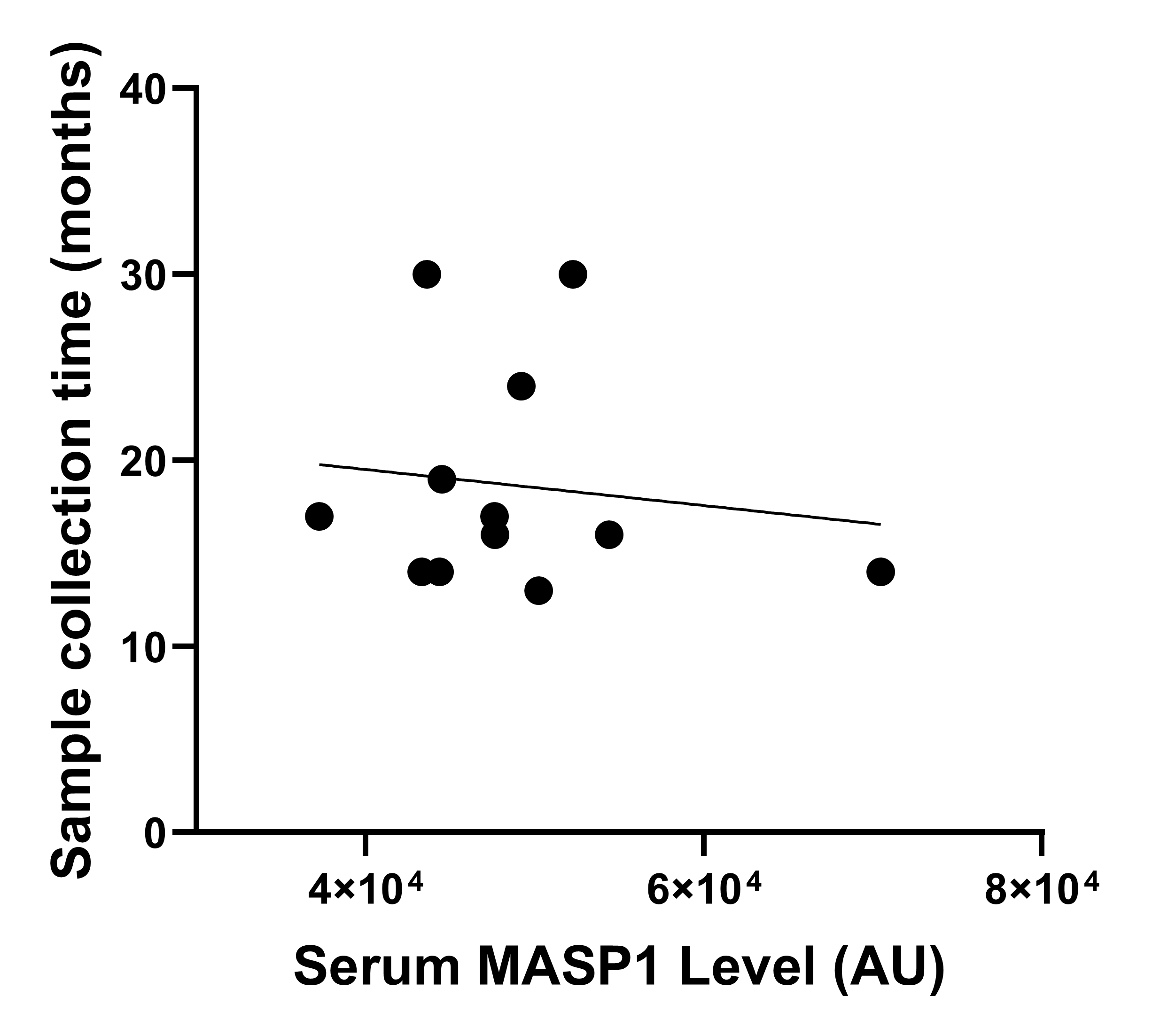

Supplement: Supplementary Figure 1 — MASP1 levels against the collection times of non-union fracture samples. [file Image1.tif]
